# Don’t let the perfect be the enemy of the good

**DOI:** 10.1371/journal.pbio.3001327

**Published:** 2021-07-15

**Authors:** Marcus Munafò

**Affiliations:** 1 MRC Integrative Epidemiology Unit at the University of Bristol, Bristol, United Kingdom; 2 School of Psychological Science, University of Bristol, Bristol, United Kingdom

## Abstract

There is growing interest in the factors that influence research quality, and into research culture more generally. Reform must be evidence-based, but experimental studies in real-world settings can be challenging. This Perspective article argues that observational evidence, even if imperfect, can be a valuable and efficient starting point to help identify the most fruitful avenues for meta-research investment.

## Teaser

There is a growing interest in the factors that influence research quality and into research culture more generally. Reform must be evidence based, but experimental studies in real-world settings can be challenging. Observational evidence, even if imperfect, can be a valuable and efficient starting point to help identify the most fruitful avenues for meta-research investment.

Concerns about the robustness of scientific research, the quality of the outputs we generate, and the incentives that shape our behaviour as scientists are not new—famously, Charles Babbage decried the state of science in general (and the Royal Society in particular) in his *Reflections on the Decline of Science in England*, *and on Some of its Causes*, published in 1833 [1]. The current debate around replicability and reproducibility needs to be understood in this context—there have always been concerns that we are not doing as good a job as we might and suggestions as to how we might do better. This is a good thing; individuals, organisations, and sectors should always take it upon themselves to reflect on these questions in the pursuit of excellence and a positive, healthy culture.

The current version of this debate can perhaps be traced back to an article published by Ioannidis in 2005, provocatively titled “Why Most Published Research Findings Are False” [2]. Meta-research—the use of the scientific method to understand science itself—certainly predates this, as does the wider discussion of whether we can do better. However, over the last 15 years, there has been a great deal of research into the question of whether published research findings are indeed robust, and, importantly, which factors contribute to this—from individual-level cognitive biases to systemic pressures and structural problems. This can perhaps be understood as the epidemiological study of the research ecosystem—trying to identify causal risk factors for adverse outcomes that are potentially amenable to intervention.

Understanding the root causes of disease can, in principle, lead to real change. For example, the causal effect of smoking on lung cancer (and many other disease outcomes) resulted in public health campaigns that have served to dramatically reduce rates of smoking in many countries, with enormous health benefits. If we similarly identify factors that influence research quality and culture, we can, in principle, intervene. Of course, the situation is not quite as simple as that—few things have as large an effect as smoking on health, whether we are talking about health outcomes or research culture. Moreover, the research ecosystem is highly interconnected—intervening to modify one process may lead to compensatory changes elsewhere or negative unintended consequences. One such example is the weaponisation of open research by financial vested interests [3].

Nevertheless, our goal should not just be to describe problems, but to solve them. To do this, we need to implement and evaluate interventions (including possible negative side effects). Excitingly, we are now starting to see exactly this—the empirical assessment of innovations that have been proposed as potential ways to improve how we train early-career scientists, how we work, and the quality of our outputs. For example, some journals have adopted badges to promote open research practices, hoping to incentivise and thereby increase specific behaviours. Indeed, an initial observational study provided grounds to be optimistic that this was the case [4], although a subsequent experimental study did not reach a similar conclusion [5]. It is possible that the experimental study was underpowered, as the confidence intervals were wide, but the jury is still out on the effect of this intervention.

This illustrates a key challenge—ideally, we would collect experimental data after manipulation of the mechanism being targeted. But real-world trials of changes to working practices and incentives are challenging and expensive, often requiring the collaboration of funders, publishers, and other organisations that may not be set up to support this kind of activity. Should we wait for experimental data before introducing changes we hope (and think), on the basis of observational data, might be successful? Should we consider observational data at all, given its well-established limitations identifying causal risk factors? Disregarding observational data risks letting the perfect be the enemy of the good. There is ample evidence, in my view, that there is room for improvement in how we train the next generations and conduct research, and many exciting and promising innovations in these areas. We cannot simply continue with the status quo while we wait for potentially unattainable perfect evidence.

What we require is a process of continuous evaluation and triangulation of evidence from different sources that bring different strengths, weaknesses, and sources of bias [6]. In this issue of *PLOS Biology*, Brandt and colleagues [7] report evidence, across 10 institutions, that providing additional training courses for PhD students intended to broaden their professional competencies was not associated with an increase in time to degree or affected their productivity, as measured by published manuscripts. This kind of analysis is prone to bias, as the authors acknowledge—for example, those selecting into this training will not necessarily be representative of the wider population of PhD students. In particular, while the authors conclude that PhD students “should participate in career and professional development opportunities that are intended to prepare them for a variety of diverse and important careers in the workforce,” what their results really show is that those who choose to do so are not negatively affected (in terms of time to completion, etc.). The evidence is not perfect; a randomised controlled trial would be better. But the work nevertheless provides valuable information.

There has never been an experimental study of whether smoking causes lung cancer—that would be unethical and impractical. But that did not prevent action as the observational evidence became overwhelming. Indeed, calls for perfect evidence and criticisms of the observational data primarily came from the tobacco industry or scientists funded by them. Any study will have limitations, and we should recognise these. But we should also be pragmatic and ensure that we are consistently updating our beliefs and activity as evidence evolves. In many cases, experimental studies are perfectly feasible (e.g., most preclinical research). In other cases, they are necessary (e.g., approval of novel pharmaceutical products or medical devices). However, in some cases, we need to recognise the limits of what is possible and use imperfect data to allow us to move forward, particularly when the risks of doing so are limited or when the change in practice is being introduced anyway (as in the case of the trainee professional development studied by Brandt and colleagues).

Ultimately, a cluster randomised trial of trainee professional development, where institutions are randomised to deliver this training or not, would provide the most compelling evidence. The study by Brandt and colleagues may serve to motivate such a study and potentially inform its design. But as changes to working practices, incentives, and research culture are introduced across the research ecosystem, we should be collecting the data we can to understand as best as we can the potential impacts—both positive and negative—of these changes.
